# Online adaptive radiotherapy and dose delivery accuracy: A retrospective analysis

**DOI:** 10.1002/acm2.14005

**Published:** 2023-04-25

**Authors:** Igor Bessieres, Olivier Lorenzo, Aurélie Bertaut, Aurélie Petitfils, Léone Aubignac, Julien Boudet

**Affiliations:** ^1^ Department of Medical Physics Centre Georges François Leclerc Dijon France; ^2^ Methodology Data‐Management and Biostatistics Unit Centre Georges‐François Leclerc Dijon France

**Keywords:** adaptive radiotherapy, dose delivery accuracy, MR‐linac, PSQA

## Abstract

**Purpose:**

With online adaptive radiotherapy (ART), patient‐specific quality assurance (PSQA) testing cannot be performed prior to delivery of the adapted treatment plan. Consequently, the dose delivery accuracy of adapted plans (i.e., the ability of the system to interpret and deliver the treatment as planned) are not initially verified. We investigated the variation in dose delivery accuracy of ART on the MRIdian 0.35 T MR‐linac (Viewray Inc., Oakwood, USA) between initial plans and their respective adapted plans, by analyzing PSQA results.

**Methods:**

We considered the two main digestive localizations treated with ART (liver and pancreas). A total of 124 PSQA results acquired with the ArcCHECK (Sun Nuclear Corporation, Melbourne, USA) multidetector system were analyzed. PSQA result variations between the initial plans and their respective adapted plans were statistically investigated and compared with the variation in MU number.

**Results:**

For the liver, limited deterioration in PSQA results was observed, and was within the limits of clinical tolerance (Initial = 98.2%, Adapted = 98.2%, *p* = 0.4503). For pancreas plans, only a few significant deteriorations extending beyond the limits of clinical tolerance were observed and were due to specific, complex anatomical configurations (Initial = 97.3%, Adapted = 96.5%, *p* = 0.0721). In parallel, we observed an influence of the increase in MU number on the PSQA results.

**Conclusion:**

We show that the dose delivery accuracy of adapted plans, in terms of PSQA results, is preserved in ART processes on the 0.35 T MR‐linac. Respecting good practices, and minimizing the increase in MU number can help to preserve the accuracy of delivery of adapted plans as compared to their respective initial plans.

## 1 INTRODUCTION

The advent of linear accelerators (linacs) with embedded magnetic resonance (MR) imaging has enabled MR‐guided radiotherapy (MRgRT) via the two commercially available systems, namely the Unity (Elekta AB, Stockholm, Sweden) and the MRIdian (Viewray Inc., Oakwood Village, OH, USA). These systems offer a 6 MV flattening‐filter‐free (FFF) linac delivering step‐and‐shoot (segmental) intensity modulated radiotherapy (IMRT) treatments, including online adaptation of treatment plans based on daily MR treatment setup scans.^1^, ^2^ Adaptive radiotherapy (ART), which considers daily modifications in organs at risk (OAR) and target volumes, makes it possible to ensure that dose constraints are respected, while achieving optimal target volume coverage.^3^ In our institution, the MRIdian has been in use since June 2019, for ART oriented toward stereotactic body radiation therapy (SBRT) of abdominal tumors, mostly of the liver and pancreas.^4^

During IMRT, the respect of good clinical practices requires the performance of patient specific quality assurance (PSQA) measurements for each treatment plan, in order to assess and validate its dose delivery accuracy.^5^ The accuracy of dose delivery can be defined as the ability of a system to interpret and deliver a treatment plan as it was generated in the treatment planning system (TPS). During online ART processes, it is not possible to include the PSQA step, because of the presence of the patient on the treatment table, and also due to time pressure.^6^, ^7^, ^8^ Consequently, the accuracy of delivery of the adapted plans cannot be verified before treatment.

Several studies have focused on the evaluation of ART processes using end‐to‐end anthropomorphic phantoms.^9^, ^10^, ^11^, ^12^, ^13^ Recently, Elter et al.^9^ evaluated the process in a realistic way using a deformable anthropomorphic pelvic phantom, with 3D dose distribution assessment through gel detectors. These studies have provided interesting evaluations of the ART process in specific cases, but did not evaluate overall adapted plan delivery accuracy.

In this context, we sought to investigate the impact of the ART process on the delivery accuracy of adapted treatment plans, in a retrospective evaluation of 3Dγ pass rates of PSQA performed after delivery of adapted plans. To this end, over 100 PSQA results from adapted plans for liver and pancreas treatments performed with the ArcCHECK (Sun Nuclear Corporation, Melbourne, USA) were analyzed, and compared to the PSQA of the initial treatment plans. Our primary objective was to verify whether the 3Dγ pass rate of adapted plans is maintained with the specific practices applied during the ART process. Indeed, the manufacturer (Viewray Inc.) recommend paying particular attention to the variation in monitor unit (MU) number during the ART process, because of its influence on the irradiation time, and possibly on the plan delivery accuracy. We therefore studied the variation in MU number between the initial and adapted plans, and compared it to the PSQA results.

To the best of our knowledge, no study to date has investigated the PSQA dilemma during the ART process, using appropriate statistical testing. For our institution, this study was a key step towards validating our clinical and dosimetric practices, in order to justify the withdrawal of adapted plan PSQA from our ART workflow.

## 2 METHODS

### 2.1 MRIdian ART process

#### 2.1.1 General description

**FIGURE 1:**
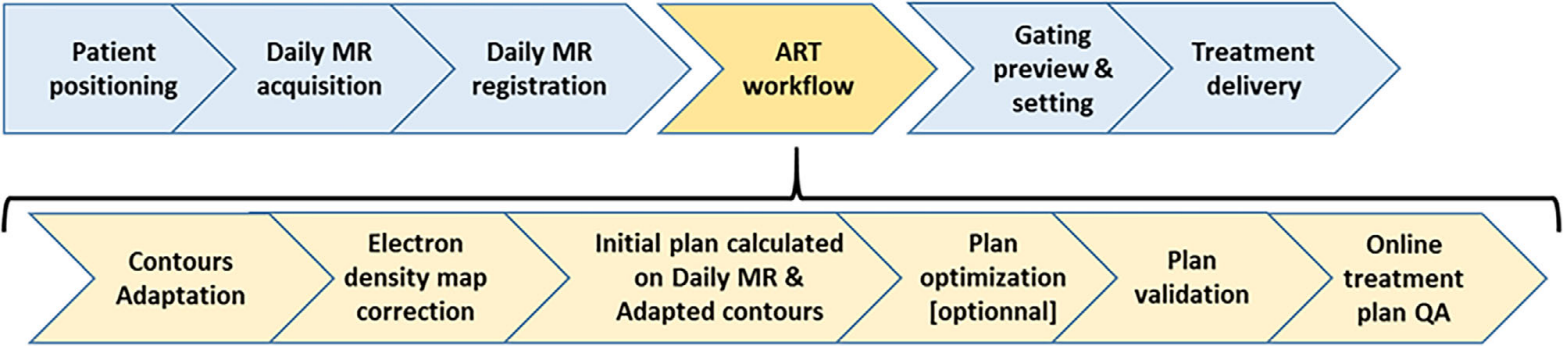
MRIdian fraction workflow: systematic steps (blue) and additional steps for ART workflow (yellow).

The MRIdian ART process is a succession of well‐defined steps that must be strictly followed, in order to ensure accurate delivery of treatment (Figure 1). At each fraction, a daily MRI scan of the patient is initially performed and registered to the primary planning image, considering the gross tumor volume (GTV) or the clinical target volume (CTV). In this study, each treatment considered was planned and delivered in a specific and reproducible breath‐hold position defined at the simulation step. In addition to the importance of patient installation, using the appropriate positioning and immobilization devices, strict control of correct breath‐holding during the daily MR acquisition is required, to reduce excessive body variations, and to limit variations in the internal structures, as compared to the primary planning image.

Then, the initial planning contours and electron density map are adapted to the daily MR image, respectively by a radiation oncologist and a physicist. The approach applied is based on that described by Bohoudi et al.^14^ consisting of limited checks and correction for daily changes within a distance of 3 cm from the planning target volume (PTV). This method assumes that this region includes the highest dose gradients, with the possible hot spot variations significantly affecting OAR doses. According to the prescribed dose, sensitive OAR initially defined by the radiation oncologist are re‐considered daily in the ART workflow. An optimized PTV is generated daily with the help of cropping rules to spare OAR according to their position with regard to the CTV. Then, the original treatment plan is recalculated using the adapted contours of the day. By comparing the daily dose distribution to the initially planned dose distribution, the radiation oncologist can choose to treat with the initial plan (i.e. no adaptation) or to adapt the treatment plan. The adapted plan is obtained by performing TPS re‐optimization, taking into account the daily optimized PTV as the target volume. In case of adaptation, the only QA available before irradiation is a secondary Monte Carlo dose calculation (for TPS calculation verification) performed with gamma index analysis. Immediately before delivering the adapted plan, the settings of the gating process are entered, and the feasibility is verified as follows: (i) delineation of the tracked volume, (ii) definition of the gating limits, adjusting the “beam on” window, and (iii) preview of the gating process on a live sagittal slice MR image.

#### 2.1.2 Online adaptation: optimization practices and MU considerations

Re‐optimization can be performed at three levels of complexity: first, simple segment‐weight optimization; second, fluence re‐optimization based on the original set of planning parameters, or third, full optimization based on modified and adapted objectives.^14^, ^15^, ^16^, ^17^, ^18^ The irradiation conditions will logically be changed, whatever the optimization choice, and in particular, the MU number.

In our institution, the initial plan settings are kept during re‐optimization, in order to limit sources of variation as much as possible, in particular the beam number with associated angles, and the maximal number of IMRT step‐and‐shoot segments that are fixed. A specific and unique maximal number of segments is set for each initial plan of each patient.

Online plan adaptation needs to be performed as quickly as possible because of the presence of the patient in the treatment position.^14^ For each step, the therapist, radiation oncologist and medical physicist have to be well trained to optimize their operating time. In this context, the re‐optimization step should not increase the delivery time, which is classically high (often around 10 min) on the MRIdian Linac with a global ART fraction duration than can exceed an hour.^19^ This issue is of paramount importance, especially for abdominal treatments, such as those considered in this study, which are delivered in the breath‐hold position. An increase in the delivery time will increase the number of apneas required to deliver the entire treatment, with the risk of tiring the patient. In this situation, repeating and reproducing the right breath‐old position could be complicated for the patient, to the detriment of target volume coverage.^20^

### 2.2 Description of the treatment plans

For this study, we included the treatment plans of 30 patients treated for abdominal tumors, namely: 15 patients treated for liver cancer and 15 patients treated for pancreatic cancer. For each patient, SBRT was prescribed and treated over a maximum of 10 days. The dose prescribed is adjusted by the radiation oncologist considering the proximity of certain OARs. This aspect is one of the main differences between these two cancer localizations, as the number of highly radiosensitive OARs is lower around the liver than around the pancreas, which is surrounded by digestive structures (duodenum, bowel, or stomach). Consequently, the level of dose prescription is often higher for liver tumors (40, 45, or 50 Gy in five fractions of 8, 9, or 10 Gy) compared to pancreatic tumors (30, 35, or 40 Gy delivered in five fractions of 6, 7, or 8 Gy). Thus, a total of 30 initial treatment plans plus their adapted fractions were considered in this study. Among the 150 treatment fractions, a total of 124 were adapted (82%), mainly due to OAR modifications and dose constraint failures. In the large majority of cases, re‐optimization was done with the original planning parameters. For liver plans, the mean and standard deviation for beam number was 15 ± 3 (median: 16, range [9–19]) and for segment number was 53 ± 9 (median: 52, range [37–76]). For pancreas plans, the mean and standard deviation for beam number was 16 ± 2 (median: 15, range [14–19]) and for segment number was 57 ± 6 (median: 59, range [49–68]).

### 2.3 Assessment of dose delivery accuracy

The dosimetric plan quality as described by Moore et al.^21^ was not evaluated in this study. We assumed that the clinical evaluation and validation of adapted plans was optimal, and equivalent between each adapted plan and its corresponding initial plan. Only the variation in the accuracy of dose delivery was considered, and defined here as a variation in PSQA 3Dγ analysis results.

To this end, in addition to the 30 PSQA measurements from the initial treatment plans, we also calculated and analyzed PSQA measurements for the 124 adapted fractions after treatment delivery. To do this, the ArcCHECK cylindrically shaped QA device was used. It is made of PMMA with an outer diameter of 26.6 cm and an inner cavity diameter of 15.1 cm. The device includes 1386 diode detectors of a size of 0.8 × 0.8 mm^2^, helically arranged at a physical depth of 2.9 cm. An MRI‐compatible device was used for this study, and previously validated on the MRIdian system.^22^ The ArcCHECK software system, called SNC Patient (version 8.4) enables comparison between the measured and planned dose, with global or local gamma index analysis. Considering the bore design of the MRIdian system and its limited diameter (70 cm), the ArcCHECK can be lateralized in order to center the device on the significant isodose and optimize the consistency of the PSQA. This process has been validated in a previous study.^23^ Systematically, the same positioning of the ArcCHECK was used for adapted plan checks, as that used for the initial plan.

Considering that this study used SBRT, gamma index pass rates were analyzed with a dose difference and distance to agreement (DTA) threshold of respectively 2% and 2 mm, with a 10% dose threshold. Firstly, global normalization was considered in the analysis for its superior clinical relevance.^5^ Nevertheless, local normalization was also analyzed because of its sensitivity to the high dose gradient often observed in SBRT treatment. Consequently, both forms of analysis are of value in characterizing the dose delivery accuracy.

The action limits (ALim) and tolerance limits (TLim) were defined according to the procedure described by Miften et al.,^5^ offering a process view including all sources of variation. The TLim is the minimum value that keeps the process unchanged. The ALim is the minimum acceptable performance value and is lower than the TLim. If a result is lower than the TLim but outside the ALim, the physicist has to determine whether or not action should be taken.^5^ For ease of use, the TLim clinically applied is the calculated TLim, rounded up to the nearest multiple of five, and thus, more restrictive. All the values are summarized in Table 1.

**TABLE 1:** Gamma index analysis limits.

	Global	Local
Clinical TLim	95.0%	85.0%
Calculated TLim	92.8%	82.0%
Calculated ALim	90.2%	72.8%

Consequently, the clinical TLim of the gamma index pass rate was set for local and global dose difference analysis at respectively 85.0% and 95.0%.

### 2.4 Influence of MU number

During initial user training, the manufacturer (Viewray Inc.) usually recommends containing and limiting the MU increase to +20% of the initial plan's MU number during the ART process. As the dose rate is constant on the MRIdian (600 MU/min^1^), the MU number is a relevant parameter to characterize the total delivery time, considering that the number of segments is unchanged. Variations in plan complexity cannot be completely characterized using only MU number variation. Nevertheless, absent other plan complexity indices, it could be of direct help for the users if a relation is established between treatment PSQA result variation and MU number variation.

**FIGURE 2:**
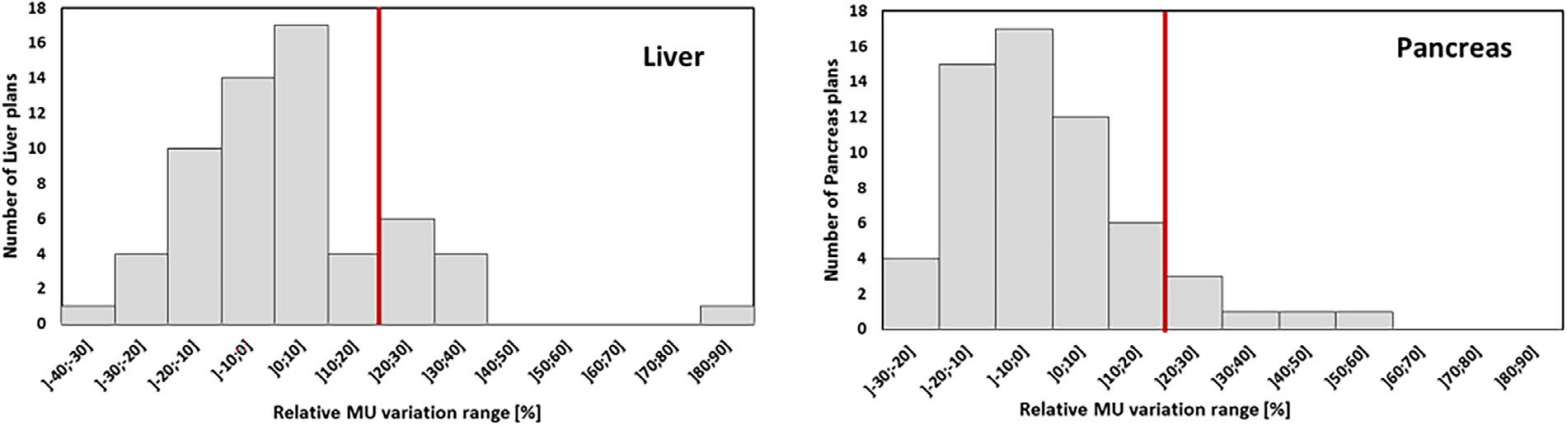
Distribution of MU relative difference between adapted plans and their respective initial plan. Liver plans are represented on the left side and Pancreas plans are represented on the right side. The red lines represent the +20% limit suggested by the manufacturer.

**FIGURE 3:**
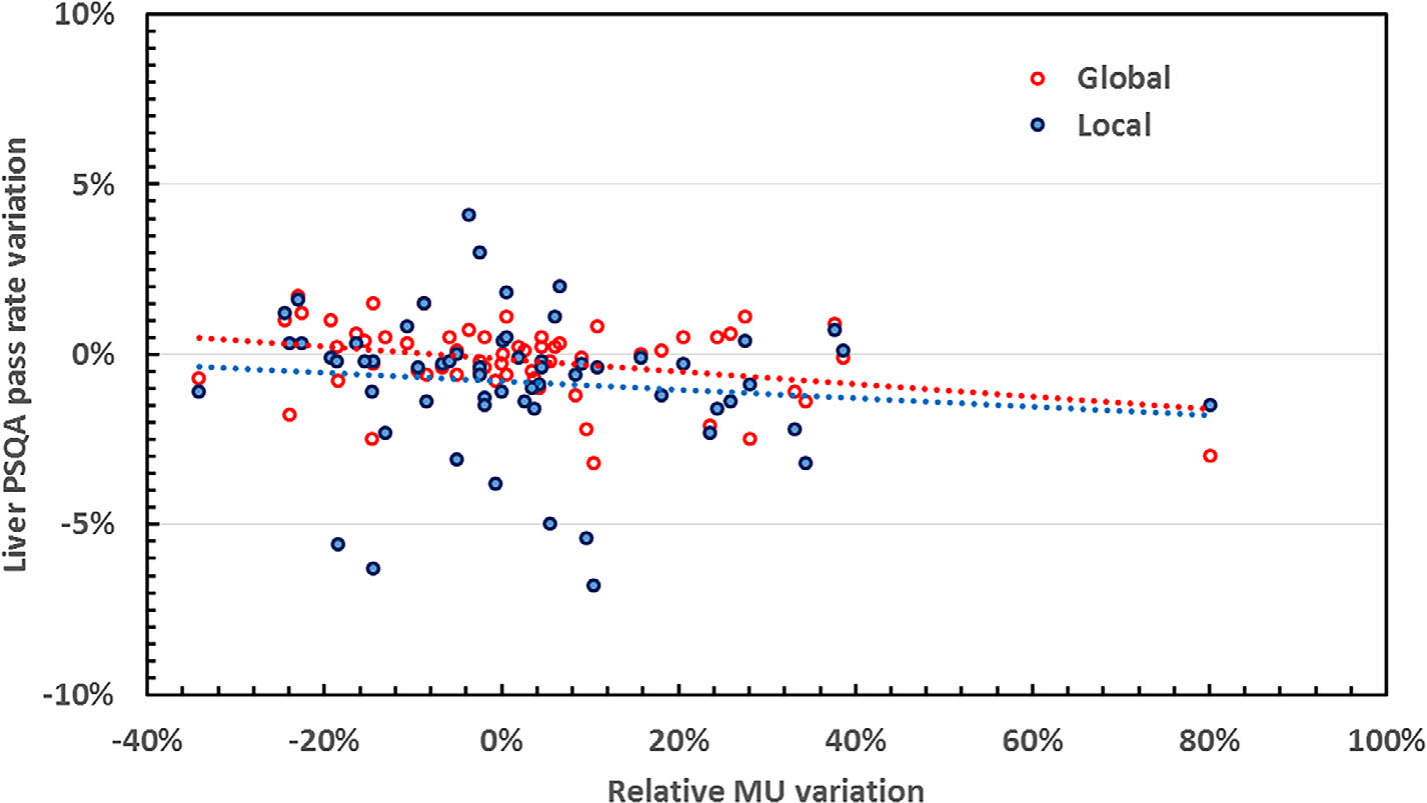
PSQA results variation for liver patient according to the MU variation between the initial and each adapted plan.

**FIGURE 4:**
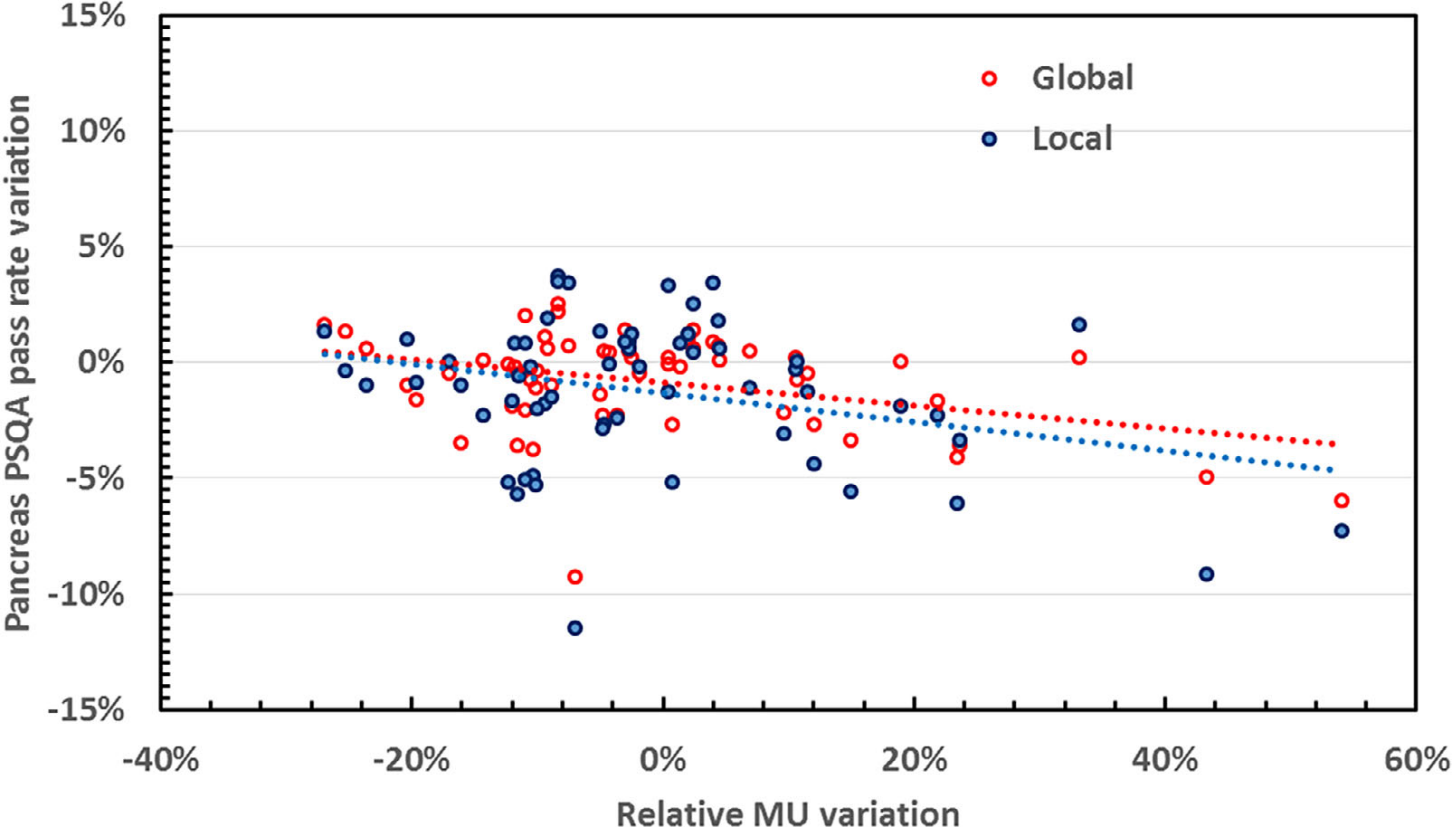
PSQA results variation for Pancreas patient according to the MU variation between the initial and each adapted plan.

In this context, variation in MU number distribution was plotted for each adapted plan (Figure 2) and then investigated to analyze our practices and their possible impact on PSQA 3Dγ analysis results. For each adapted plan, differences in PSQA pass rates between the initial plan and the adapted plan were calculated and plotted according to relative variation in MU number (Figures 3 and 4).

### 2.5 Statistical analysis

PSQA pass rates are described as mean values with standard deviation (SD). Medians and range were also calculated. The non‐parametric Wilcoxon signed rank test was used to assess the difference between the mean values of initial versus adapted PSQA. Correlations between PSQA pass rate variations and relative MU variations were tested using Pearson's correlation coefficient. All tests were two‐sided, and *p*‐values < 0.05 were considered statistically significant. All analyses were performed using SAS version 9.4 (SAS Institute Inc., Cary, NC, USA).

## 3 RESULTS

### 3.1 Characterization of dose delivery accuracy

#### 3.1.1 Global approach

In a first approach, PSQA results were considered for each localization (liver and pancreas). Table 2 presents the means and medians of local and global initial and adapted gamma index pass rates for all patients.

**TABLE 2:** Global and local initial and adapted PSQA ArcCHECK gamma pass rates for the liver and pancreas.

2%/2 mm, Th. 10%	Liver		Pancreas	
	Global	Local	Global	Local
Mean initial pass rate (*n*_Liver_ = 15 and *n*_Pancreas_ = 15)	**98.2% ± 1.1%**Median = 98.3% [95.1%, 99.8%]	**95.0% ± 2.5%**Median = 95.1% [90.6%, 98.4%]	**97.3% ± 0.9%**Median = 97.3% [95.6%, 99.3%]	**93.9% ± 2.0%**Median = 94.3% [90.5%, 97.2%]
Mean adapted pass rate (*n*_Liver_ = 61 and *n*_Pancreas_ = 63)	**98.2% ± 1.4%**Median = 98.6% [94.3%, 100.0%]	**94.3% ± 3.3%**Median = 95.3% [85.0%, 98.3%]	**96.5% ± 2.6%**Median = 96.9% [87.5%, 100.0%]	**92.4% ± 3.6%**Median = 93.4% [82.1%, 98.1%]
Pass rate variation (mean initial—mean adapted)	0.0%	−0.7%	−0.8%	−1.5%
Wilcoxon rank test *p*‐value	0.4503	0.0015	0.0721	0.0028

Values are presented as mean ± Standard deviation, and Median [Range].

**For liver and pancreas cancer patients**, there was no significant difference in global mean value whereas there was a significant difference in local mean value.

Consequently, no significant impairment of PSQA results was observed based on the global analysis. Nevertheless, based on local analysis, there was a significant degradation in 3Dγ results between the initial and the adapted PSQA. The degradation was greater for pancreas plans than for liver plans (−0.7% vs. −1.5%). The mean values were within the TLim (85.0% for local pass rate and 95.0% for global pass rate) for each group.

#### 3.1.2 Detailed analysis

Table 3 lists the number of PSQA results among the adapted plans that did not satisfy the clinical TLim.

**TABLE 3:** Number of adapted plans with PSQA results outside the clinical TLim.

	Liver (61 adapted fractions)	Pancreas (63 adapted fractions)
Global (<95%)	3	14
Local (<85%)	0	3

**For liver plans**, only three global PSQA results of the adapted plans fell outside the clinical TLim, with a limited decrease in the pass rate: 94.8% for two plans and 94.6% for one. All the local analysis PSQA results satisfied the clinical TLim of 85.0%. Consequently, the PSQA results of all the adapted liver plans were clinically acceptable and validated.

**For pancreas plans**, 14 global PSQA results from the adapted plans failed to satisfy the clinical TLim, accounting for around 22% of all fractions. For local analysis PSQA results, the clinical TLim was not satisfied for three adapted plans, whereas the calculated TLim was satisfied.

Unlike liver plans, the PSQA results of several adapted plans for the pancreas were impacted, with 14 failed plans according to the global analysis. Nevertheless, 10 of these 14 plans were in agreement with the calculated TLim (92.8%).

Finally four plans did not satisfy the clinical TLim for the global analysis. Nevertheless, for two of these four plans, the impairment to PSQA results was limited, and the global pass rates (respectively 90.6% and 92.2%) were above the ALim (90.2%). For the two most substantially deteriorated adapted plans, global pass rates outside the ALim were observed, at respectively 87.5% and 89.6%, accounting for 1.6% of all adapted plans included in this study.

### 3.2 Influence of variations in MU on PSQA results

MU number variation was analyzed because of its relation to the time pressure of the ART process, and because of the possible impact of an increase in MU number on the complexity and dose delivery accuracy of the plan.

First, we calculated the variation in MU between the initial and the adapted plan, for both localizations. The distribution of the differences is shown in Figure 2.

For most plans, the increase in MU number was contained, and within the maximum limit of +20% recommended by the manufacturer. For some plans, an increase of the MU number beyond the +20% limit, and beyond +40% in a small number of cases, was observed for both types of tumor. Considering these distributions and the high number of substantial variations in MU number, we investigated the influence on PSQA results. To this end, the variation in PSQA results as a function of the variation in MU number for each adapted plan was calculated and is plotted in Figures 3 and 4, respectively for liver and pancreas global and local analysis. Pearson's correlation coefficients for each group are detailed in Table 4.

**TABLE 4:** Pearson correlation between the PSQA results variation and MU variation.

		Pearson correlation coefficient *ρ*	*p* value
Liver	Global	−0.32797	0.0099
	Local	−0.11699	0.3692
Pancreas	Global	−0.36394	0.0043
	Local	−0.30769	0.0168

For liver global analysis, there was a significant inverse correlation between PSQA result variation and MU variation, whereas there was no statistically significant correlation between liver local analysis PSQA results and MU variation. For the pancreas, for both global and local analysis, there was a statistically significant inverse correlation between PSQA result variation and MU variation (Table 4).

Overall, with the exception of liver local analysis PSQA results, we noted a significant deterioration in PSQA results with increasing MU number. These results were confirmed by the results of the linear regression, showing a linear trend (Figures 3 and 4).

## 4 DISCUSSION

PSQA results of the adapted plans mostly showed limited degradation of dose delivery accuracy. In particular, for liver plans, the level of PSQA results remained within the limits of clinical tolerance in all cases. For pancreas adapted plans, some significant deteriorations in PSQA results were observed, but corresponded to complex configurations. Indeed, the four most impacted plans were from patients treated for synchronous double lesions situated in close proximity to one another (distance <5 cm).^4^ These synchronous treatments are complex, in particular because of the limitation of the dose contribution between both plans. Consequently, according to the accuracy of the repositioning of the patient and the modification of the OAR position or the distance between the two lesions, re‐optimization during the adaptive process could increase the plan complexity in terms of MU number or segment shapes. This likely explains why deteriorations in PSQA pass rates were mainly observed in these patients.

Intuitively, the more OARs that are associated with the ART process, the harder it is to plan the treatment. For most liver plans, only one or two OARs were included in the ART process, whereas up to five OARs had to be taken into consideration for pancreas plans. This anatomical configuration logically influences the accuracy of dose delivery in ART. Nevertheless, severe degradations beyond the ALim were only observed for two pancreas plans. For one of these, the increase in MU number was >50%, and was attributed to a sudden change of the optimization parameters by the physicist. When we reviewed this plan, we deemed that the change was not imperative and should have been avoided in order to remain in compliance with good practices. Moreover, this change occurred at the beginning of our experience with the ART process, explaining why we did not find an optimal alternative to change this MU deviation. Considering the good target coverage and the respect of the dose constraints to the OAR, the plan was delivered nonetheless. The change in dose delivery accuracy was pointed out after the delivery with PSQA measurements. With our experience, we would not have treated this plan now.

Because of the increased delivery time and the reduced dose delivery accuracy, it is important to follow good practices by carefully and gradually changing the optimization settings. The variation in MU should be systematically evaluated prior to plan delivery. Indeed, in addition to tiring the patient, we observed clear evidence of a tendency for the dose delivery accuracy of the plan to decrease with increasing MU number.

**FIGURE 5:**
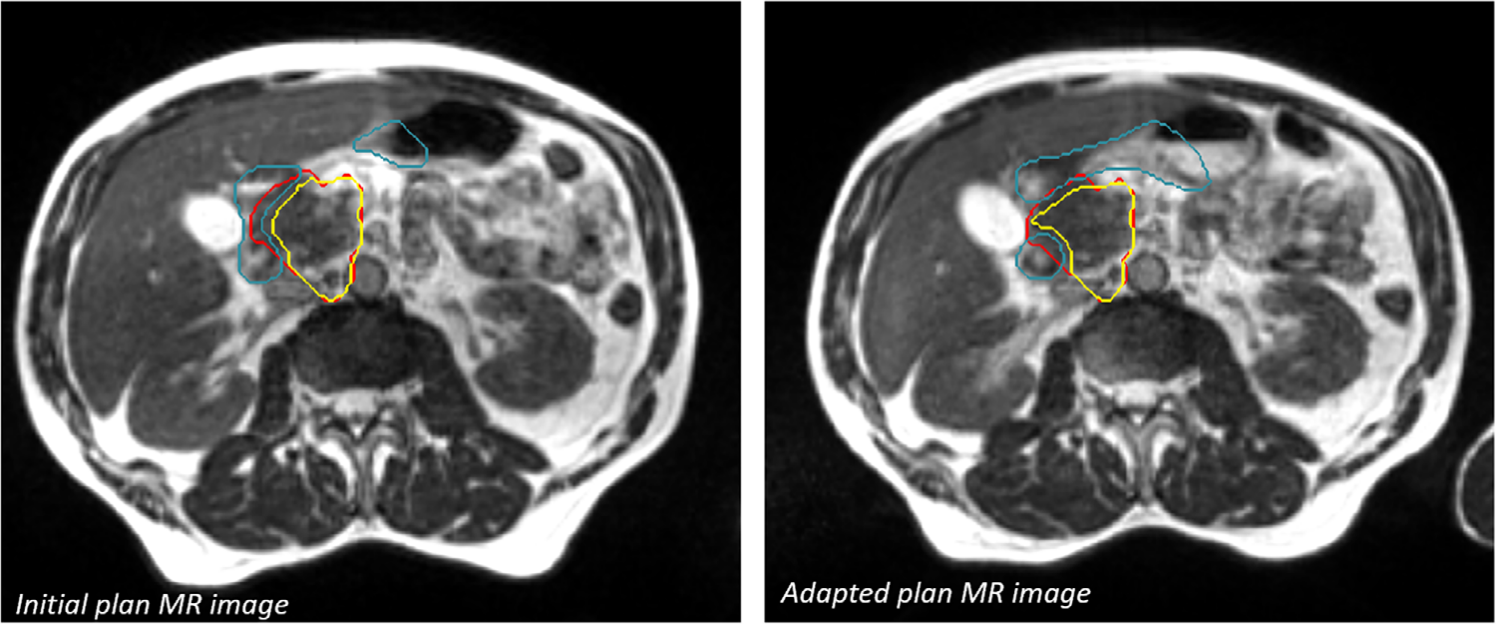
Variation of OAR shape (Duodenum in blue) between initial and adapted fraction with the influence on the optimized PTV shape (in yellow). The original PTV is in red.

Although it is in line with good practices to keep the beam configuration and optimization parameters unchanged, the MU number may increase significantly. Indeed, as illustrated in Figure 5, changes in the shape or position of OARs can impact on target volume shrinkage, and consequently, on the complexity of the target volume shape. Furthermore, a reduction in the distance between the OAR and the target volume can require a steeper dose gradient to satisfy dose constraints. These configurations may lead to more complex planning, with a resultant significant increase in MU.

In light of the results of this study, our institution decided to stop performing adapted fraction PSQA after delivery. Even if an initial plan may potentially never proceed to treatment delivery, it still has to be verified, to check the constancy in our dosimetric practices, and in order to use it as a predictive index for the dose delivery accuracy of adapted plans. The present study was performed on only two cancer sites, but the decision to stop PSQA was made for all adapted localizations, on the assumption that the pancreas is the most complicated and sensitive localization and we follow the same practices for each site. To the best of our knowledge, most MRIdian users do not perform PSQA measurements for their adapted plans, because it is very time consuming. For those who perform post‐delivery PSQA measurements, our investigation describes a means to discontinue PSQA measurements without changing the quality assurance process. It should be noted however that this work was validated only for two localizations, and within the specific conditions of planning, adapting, and measuring implemented in our study. Further validation is required, especially for users working in different conditions, before stopping adapted plan PSQA measurements.

One limitation of this study is that the dose delivery accuracy was assessed solely on the basis of PSQA measurement obtained with the ArcCHECK, which itself presents several limitations to its performance that may have impacted our results.^24^ To be more comprehensive, delivery accuracy and complexity of a treatment plan could be defined in terms of other aspects, including the Multileaf collimator (MLC) field shape or using modulation indices.^25^ In the context of step‐and‐shoot IMRT, the investigation of the size of the MLC segment weighted by the number of MU might be of value for assessing the impact on dose delivery accuracy. In this regard, Lamb et al.^18^ established online automatic plan consistency checks for Viewray's system based on the evaluation of two parameters: first, the ratio of the “bixel‐minutes,” defined as the sum of beam on time multiplied by segment area (a measure of integral dose); and second, the target volume ratio are evaluated as safety checks. This solution makes it possible to monitor the quality of adapted fractions without being too time consuming. More recently, Rippke et al. similarly developed an automatic tool for process‐based per‐fraction QA for online ART on the MRIdian Linac based on the plan analysis.^26^ This type of dosimetric checking has also been investigated on the Unity (Elekta AB, Stockholm, Sweden) MR linac system.^27^ In terms of perspectives for future studies, it would be of interest to compare the results of these different tools with the results of PSQA measurement, with a view to investigating a possible correlation.

Three additional QA processes may also be warranted to monitor the quality and delivery of treatment plans and, more specifically, on‐table adapted treatment plans. These processes are: independent dose calculation, in vivo dosimetry and logfile QA. These three QA processes could be automatized and applied in the workflow without significantly increasing the workload. Indeed, before delivery, independent dose calculation should make it possible to verify whether dose calculation and MU are in agreement. This function is already commercially available on the Viewray and Elekta MR linac systems.^28^ After delivery, logfile analysis could inform the user about whether the machine has performed the treatment as planned based on machine parameters. This solution is built‐in for the Viewray system. On Elekta MR linac systems, logfiles can be read out and recorded for dose reconstruction.^29^ Also after delivery, in vivo dosimetry could help to check whether the treatment went as planned, by taking into account the presence of the patient. Currently, no commercial solution is available for this on the Viewray system. On the Elekta system, the presence of an MV imaging system should make it possible^30^ but it is still not commercially available.

More generally, the entire ART process is a highly interdisciplinary workflow with the patient in the treatment position, and consequently, is highly time sensitive. Workflows and processes need to be standardized and analyzed to identify any specific risks introduced by the ART process.^31^, ^32^, ^33^ Prospective approaches such as failure modes and effects analysis (FMEA) could be applied to quantify risks and associated failures. They may lead to the definition of appropriate process‐based QA strategies and tools that could be implemented to reduce risk and avoid critical failures.^31^, ^32^, ^33^ Conserving the quality and dose delivery accuracy of adapted plans is necessary, and implies the successful achievement of each successive step in the ART process.

## 5 CONCLUSION

In this study, post‐fraction PSQA results from adapted plans for the treatment of liver and pancreas cancer, using the MRIdian 0.35 T MR‐linac system, were investigated and demonstrate that the dose delivery accuracy of adapt plans is conserved with limited deterioration of the PSQA results. In the vast majority of cases, pass rates were within tolerance limits. The only degradation in PSQA that was outside the tolerance limits was observed for highly specific and complex treatment plans. We show that minimizing the increase in MU number between initial and adapted plans is key to maintaining the accuracy of dose delivery. In our clinical routine practice, based on the present results, we have decided to stop adapted PSQA measurement. On condition that good practices are adhered to during the ART workflow, we can assume that the results of initial PSQA measurements are comparable to those of adapted plans. Overall, the ART process is a multidisciplinary and complex process that needs to be globally analyzed to limit risks and minimize quality deviation.

## AUTHOR CONTRIBUTIONS

Igor Bessieres: Conception and design of the work; acquisition, analysis and interpretation of data for the work; drafting the work; final approval of the version to be published. Olivier Lorenzo: Conception and design of the work; analysis of data for the work; revising the work critically for important intellectual content; final approval of the version to be published. Aurélie Bertaut: Statistical Analysis and interpretation of data for the work; revising the work critically for important intellectual content. Aurélie Petitfils: Analysis and interpretation of data for the work; revising the work critically for important intellectual content. Léone Aubignac: Analysis and interpretation of data for the work; revising the work critically for important intellectual content. Julien Boudet: Conception and design of the work; analysis of data for the work; revising the work critically for important intellectual content; final approval of the version to be published.

## CONFLICT OF INTEREST STATEMENT

The authors declare no conflicts of interest
